# Population dynamics and host reactions in young foxes following experimental infection with the minute intestinal fluke, *Haplorchis pumilio*

**DOI:** 10.1186/1756-3305-6-4

**Published:** 2013-01-04

**Authors:** Sofie Nissen, Stig Milan Thamsborg, Per Walther Kania, Páll S Leifsson, Anders Dalsgaard, Maria Vang Johansen

**Affiliations:** 1 Department of Veterinary Disease Biology, Faculty of Health and Medical Sciences, University of Copenhagen, Groennegaardsvej 15, Frederiksberg C DK-1870, Denmark

**Keywords:** *Haplorchis pumilio*, Heterophyidae, Fish-borne zoonotic trematode, Minute intestinal fluke, Clinical pathology, Anaemia, Anorexia, Worm burden, Faecal egg counts, Fecundity

## Abstract

**Background:**

Infections with fish-borne zoonotic trematodes (FZT) including the minute intestinal fluke, *Haplorchis pumilio*, are highly prevalent in Southeast Asia. However, little is known about the infection dynamics and clinical symptoms in the final hosts which include a range of animal species and man. We aimed to generate such information using an experimental model with *H. pumilio* in foxes.

**Method:**

Eight commercially bred foxes were each orally infected with 2000 *H. pumilio* metacercariae. Another three foxes served as uninfected controls. Faecal examination for eggs was performed twice weekly. The body weight was measured, standard haematological and biochemical analysis were performed regularly. All foxes were euthanized at day 56 post infection (p.i.). Adult worms were quantified and location in the small intestine noted.

**Results:**

Anorexia was observed in all infected foxes starting day 12 p.i. and lasting for approximately a week. A weight loss was noticed in the infected group in weeks 3–6 p.i. Five of eight infected foxes excreted *H. pumilio* eggs day 9 p.i. onwards, the remaining three started on day 13 p.i. Mean (± SD) faecal egg counts showed an initial peak at day 16–20 with a maximum of 1443 ± 1176 eggs per gram of faeces (epg), where after a stable egg output around 4–500 epg was seen. Worm burdens ranged between 116–2070 adult flukes with a mean (± SD) worm recovery of 948 ± 666. The majority of worms were found in the lower part of the jejunum. Total white blood cell and lymphocyte counts were significant lower in the infected group from first week p.i. onwards and throughout the study period. A significantly lower level of eosinophils was found in week 2 p.i. and transient anaemia was seen in week 2 and 4 p.i.

**Conclusion:**

This study showed a short prepatency period, an initial peak in egg excretion, establishment of infection in all animals with predilection site in the lower jejunum and a marked but transient clinical effect of the infection. The findings on egg output and prepatency should be taken into consideration when control programs targeting dogs and other reservoir hosts are to be established.

## Background

Fish-borne zoonotic trematode (FZT) infections being liver- and minute intestinal flukes have in the last decades attracted increased attention in the public and research communities. In 2002, WHO listed the liver flukes *Opisthorchis viverrini* and *Clonorchis sinensis* and the intestinal heterophyids, *Haplorchis taichui* and *Metagonimus yokogawai* among the foodborne trematodes of medical importance and public health concern in Asia [1]. While the liver flukes have received more attention, less knowledge is available about the life cycle and clinical importance of the large group of more than 35 zoonotic minute intestinal trematodes, most of them belonging to the family Heterophyidae [2]. A reason for this is the tendency in surveys to overlook the intestinal flukes due to their eggs’ resemblance with liver flukes eggs [3]. However, several recent studies have assessed the prevalence, species composition, status and risk of fish-borne zoonotic parasites in Vietnamese aquaculture [4,5], humans [6], and domestic animals [7,8] and have found *Haplorchis pumilio* to be particularly prevalent [4,6,7]. Domestic animals including pigs, cats and especially dogs play an important role in sustaining the transmission by shedding heterophyid eggs into the environment [7]. In spite of the increased attention to FZT parasites, little is known about the fundamental population dynamics of the most common intestinal flukes [9,10]. Knowledge about e.g. egg excretion is needed for recommending the proper diagnostic tools [10] and for planning effective prevention and control strategies, e.g. drug treatment of reservoir hosts.

Minute intestinal trematodes infections, including *H. pumilio* infections, are generally described as asymptomatic or mild and transient unless individuals are heavily infected or immunocompromised [11]. Occasionally, humans suffer symptoms like weakness, discomfort, abdominal pain, diarrhoea, and vomiting as a result of infection with e.g. *H. taichui*[12,13]*.* Clinical symptoms of heterophyid infections are thus unspecific disregarding the species involved [2]. However, in areas where people suffer from mixed parasitic infections with both soil transmitted helminths and fish-borne zoonotic trematodes like Lao PDR [14] or Vietnam [15], it is possible that clinical effects of minute intestinal trematode infections are masked by symptoms of co-infections and that these infections have thus been neglected. Controlled studies of the host reactions towards single species infections with minute intestinal trematodes are needed to clarify the pathogenicity of these infections.

This study aimed at describing the dynamics of the infection by determining the time till patency of infection, egg excretion patterns, proportion of metacercariae that establish infection, and location of the flukes in the intestine during an experimental infection with a single administered *H. pumilio* dose. The study also aimed at assessing the pathogenicity of a single *H. pumilio* infection by determining clinical, haematological, biochemical, and pathological changes. Dogs are known to be important natural hosts of fish-borne trematodes [7]. However, for practical reasons, foxes, belonging to the same family as dogs (Canidae) were chosen as model due to their resemblance to dogs and their ability to host a range of fish-borne intestinal trematodes from the family Heterophyidae including *H. heterophyes, Cryptocotyle lingua* and *Apophallus donicus*[16-18].

## Methods

### Animals and treatment

Eleven female foxes (*Vulpes vulpes)*, five months of age were obtained from a commercial Danish breeder. The foxes were housed in groups of two or three animals in concrete pens at the experimental facilities in Taastrup, Denmark and allowed to acclimatize for 8 days. Prior to onset of the experiment foxes received prophylactic treatment for possible undetected helminths with fenbendazole (Panacur®, Intervet/Schering-Plough, 50 mg/kg body weight (bw) for 3 consecutive days) and praziquantel/emodepside (Profender® Spot-on, Bayer HealthCare, 96 mg praziquantel and 24 mg emodepside topically applied). Faecal samples were examined for eggs and larvae before and after treatment by McMaster, Kato Katz and Baermann techniques [19-21]. Until day 15 post infection (p.i) the foxes were fed daily 100–120 g of a commercial dry feed (Hill’s Science Plan Puppy Healthy Development Large Breed™ for canines, Kruuse, Denmark). Due to problems with the consistency of faeces (fatty, grainy texture) and difficulties performing the faecal examination, the diet was changed day 15 onwards to 300–330 g/day commercial wet feed. The feed contained chicken, fish and plant material and had a metabolisable energy content of 1900 kcal/kg feed. Water was provided *ad libitum*. The experiment was carried out with permission from the Danish authorities (license no. 2008/561 − 1461).

### Metacercariae

Eight foxes were each fed infected minced raw fish containing 2000 metacercariae (day 0). Three foxes were kept as uninfected controls. The source of metacercariae was infected gold fish (*Carassius auratus*) kept in the laboratory and experimentally infected with cercariae of the parapleurolophocercous type which includes *H. pumilio*, from naturally infected snails (*Melanoides tuberculata*) collected and shipped from the Nam Dinh province in Vietnam. Sixty infected gold fish (a total of 780 g) were killed by decapitation, homogenized by an immersion blender and divided into individual doses. Three sub-samples (mean 13 g/sample = 5% of total dose) of the mixture were checked by an artificial digestion using 1% pepsin solution [22] to recover and determine the density of metacercariae in the minced fish to be used for infection. The material to be used for infection was kept at 5°C prior to administration to the foxes within 24 h after preparation.

### Clinical and faecal examinations

Foxes were weighed weekly except week 1 p.i. The appetite and well-being were monitored at the daily feeding. Faecal samples were collected from the floor after separating the foxes overnight. Total amount of faeces was collected over a ten day period between day 34 – 46 p.i. to estimate the daily faeces excretion per fox. Consistency of faecal samples was scored using Walthams faecal scoring system for dog faeces [23] where 1 is hard, dry pellets, 4.5 is diarrhoea and 5 is watery diarrhoea. Faecal examination for eggs was done twice weekly by the Kato Katz method [21]. A single slide was prepared from each sample. Eggs were counted at 100× magnification. Egg counts were multiplied by 24 and expressed as eggs per gram of faeces (epg).

### Haematology and biochemical analysis

Blood samples were taken every fortnight from the jugular vein, and standard haematology (total white blood cell count (WBC), leukocyte differential count, total red blood cell count (RBC), haemoglobin, haematocrit, platelet counts), and a range of biochemical parameters were measured in serum (albumin, glucose, total protein, alkaline phosphotase, alanine aminotransaminase (ALT), total bilirubin, fructosamine, cholesterol, creatinine, phosphate, bile acid, amylase, blood urea nitrogen, gamma-glutamyltransferase (GGT), calcium, magnesium, sodium, potassium) using an ADVIA2120 hematology analyzer (Siemens) using canine settings and an automated spectrophotometer (ADVIA 1800, Siemens), respectively.

### Post mortem examinations

At day 56 p.i. all foxes were euthanized. The foxes were sedated by Zoletil/Dormitor (0.1 ml/5kg) by an intra-muscular injection and euthanized by an overdose of 20% pentobarbital (0.25ml/kg) administered intra-cardially. The abdomen of the foxes were opened longitudinally, the intestines detached and the small intestines were removed and divided into four sections: duodenum (15 cm distal of the stomach), upper part of jejunum, lower part of jejunum, ileum + caecum. Each section was placed in individual buckets with saline (37°C), opened longitudinally, and the intestinal mucosa was gently washed. The mucosa was examined macroscopically for pathological changes. A tissue sample was cut for histopathology from the jejunum (3/4 way down) after washing, fixed in 10% formalin and transferred to 70% ethanol after 7 months in formalin. Tissues were processed using routine techniques and 4–5 μm sections were cut and stained with haematoxylin and eosin (HE-staining). The livers were cut in pieces of approximately 0.5 cm, flushed with a water hose through a 500 μm sieve and examined macroscopically for liver flukes. The content from each section of the intestines was filtrated through a sieve (mesh size: 1mm) and worms were collected on two sieves (mesh size: 90 μm/38 μm) placed on top of each other. The rinsed intestinal sections were incubated in 0.9% saline at 37°C while shaken at 150 rpm. After 1 h the intestines were sub-merged in 0.9% saline with 10mM EDTA and incubated for 6 h at 37°C. After 6 h the intestine were transferred to a new container with similar EDTA concentration and temperature and incubated for another 18 h. After the last incubation the intestinal mucosa was washed using a water hose. The loosened material and all other fluids from the incubations were washed through two sieves on top of each other (mesh size: 90 μm/38 μm) and the remains from each sieve was collected in a 50 ml tube and stored in 70% ethanol. Intestinal flukes were recovered using a dissecting microscope (25 – 40×). All isolated flukes were counted, pooled and stored in 70% ethanol for later staining and species confirmation by morphology and PCR. Measurements of length and width (widest place on the worms or egg) of 83 worms and 160 eggs from the uterus from 16 worms were performed. The total number of eggs within the uterus of worms was counted for 40 worms. An equal amount of worms from all foxes were included in the measurements.

### Species confirmation

Subsamples of 56 ± 13 worms/fox (mean ± SD) were stained in Mayers haematoxylin to confirm the worm species based on morphological keys from Pearson and OwYang [24]. PCR was performed on 9 worms after isolation of genomic DNA done according to Skov *et al.*[25]. PCR was done in 60 μl reactions using 5 μl of the obtained crude lysates, 1 μM of forward primer, 1 μM of reverse primer, 1 mM of dNTPs, 1.5 mM MgCl_2_ and 1 unit of Biotaq™ DNA Polymerase (Bioline, DNA Technology A/S, Aarhus, Denmark) in the NH4 reaction buffer. The forward primer was Fluke_ITS_F3 (5^′^ CTC GGC TCG TGT GTC GAT GAT 3^′^) and the reverse primer was Fluke_ITS_R1 (5^′^ GCA TGC TTA ART TCA GCG GGT A 3^′^). A touch down PCR protocol was used in which the annealing temperature during the first 15 cycles was gradually decreased (2 cycles at 67°C, 2 cycles at 65°C, 2 cycles at 64°C, 3 cycles at 63°C, 3 cycles at 62°C, 3 cycles at 61°C) followed by 25 cycles using the final annealing temperature of 60°C. The PCR began with an initial pre-denaturation step at 95°C for 5 min; followed by 40 cycles of denaturation at 95°C for 30 sec, annealing for 30 sec, elongation at 72°C for 30 sec; and ending with a post elongation step at 72°C for 7 min. Aliquots of 5 μl of the products were analysed by 2% agarose gel electrophoresis and visualized by ethidium bromide staining. PCR products were purified using Illustra GFX PCR DNA and Gel Purification Kit (GE Healthcare, Brondby, Denmark) according to the manufacturer’s instructions. PCR products were then sequenced at Macrogen, Seoul, South Korea using the PCR primers. All reagents were from Sigma-Aldrich, Denmark if not stated otherwise.

### Calculations and statistical analysis

The daily egg production per worm was calculated as the mean epg for each fox (day 34–44 p.i.) multiplied by the mean daily faeces excretion divided by the total number of worms recovered at the time of necropsy. The haematological and biochemical parameters were analysed by an ANOVA with random effect of fox and full factorial design of the fixed effects infection (infection vs. control) and time p.i. (week 2, 4, 6, 8). The measurements on day 0 were used as covariates. Reference ranges (means ± 2SD) were calculated by using day 0 measurements for all 11 foxes. The response variable, body weight, was analysed by an ANOVA with time p. i. (week 2, 3, 4, 5, 6, 7, 8) as repeated measurements and infection (infection vs. control) as fixed effect and random effect of fox. The body weight at time zero was used as covariate. All data sets were tested for normality and variance homogeneity. Log transformation was necessary for eosinophils, monocytes, ALT and total bilirubin. Backwards model reduction was done on a 5% significance level. Post hoc tests were performed for significant effects using a Tukey correction for multiple comparisons. Two different analyses were carried out using worm counts as the response variables. The location in the intestine (mucosa or content) was analysed by a Student’s *t*-Test for paired samples. Worms recovered in the saline and the two EDTA incubations were grouped together in the analysis as recovered in the mucosa. The effect of the second explanatory variable; section in the intestine (duodenum, upper jejunum, lower jejunum, ileum + caecum) was analysed by a Poisson regression with over-dispersion using section and fox as fixed effects. A non-parametric Spearman’s correlation test was performed to analyse the relationship between worm burden, total faecal egg excretion and body weight gain (body weight, week 8 – body weight, week 0).

## Results

### Parasitological findings

**Figure 1:**
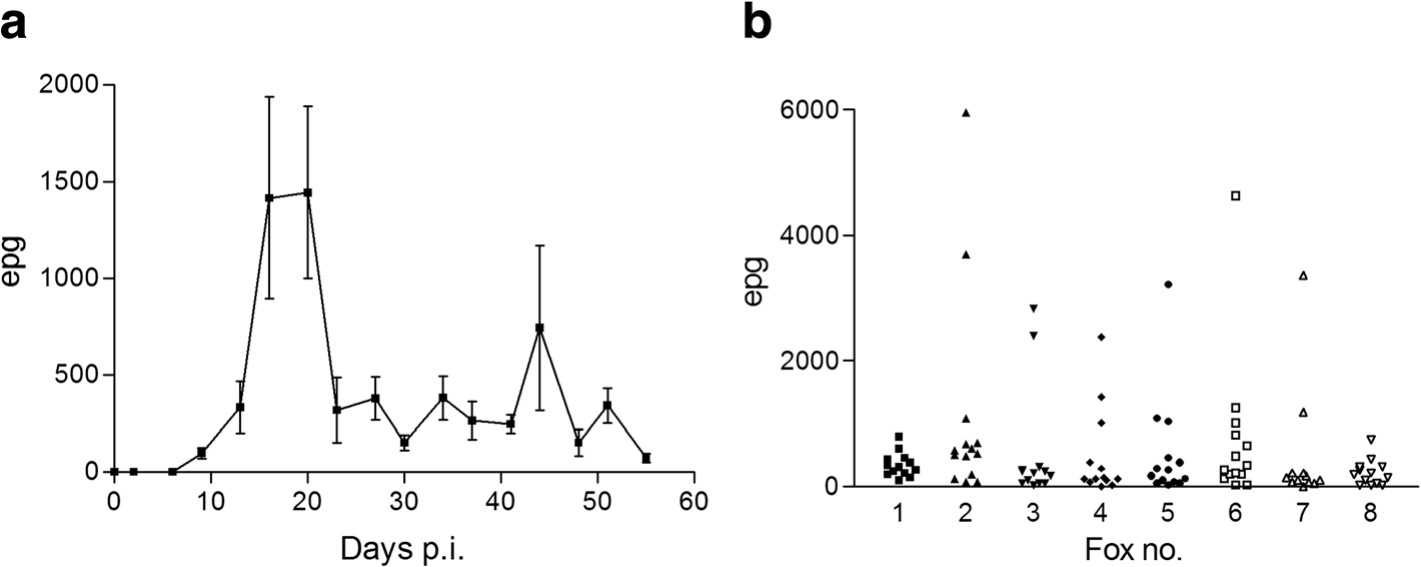
**Faecal egg excretion. **Excretion of *Haplorchis pumilio *eggs per gram of faeces (epg) from day 0–55 post infection (p.i.) from eight foxes each infected with 2000 metacercariae **a**) mean ± SD **b**) Scatter plot showing the variation for each fox over time (day 9–55 p.i.).

No helminth eggs or larvae were detected in the foxes prior to infection. Five foxes started excreting eggs day 9 p.i. and all infected foxes were excreting eggs day 13 p.i. The faecal egg counts were below 500 epg at the first two sampling days after the infection became patent, then peaked at day 16 and 20 p.i. (mean (± SD): 2598 ± 1948 epg and 1443 ± 1176 epg, respectively) thereafter a more or less stable mean egg count (< 500 epg) was seen during the rest of the study period. A peak was seen at day 44 and was due to a single fox with an egg count of 3696 epg. The egg excretion pattern over time can be seen in Figure 1a and the individual variation for each fox in Figure 1b. Mean number of eggs excreted daily per worm was calculated to be 58 ± 40. Morphometric measurements of adult worms and eggs and counts of number of eggs in the uterus can be seen in Table 1. No correlation was found between the total faecal egg excretion (whole study period) and worm burden at necropsy.

**Table 1:** Morphometric measurements

	**Number of specimens**	**Mean**	**Confidence Interval (95%)**	**Minimum – maximum**
Adult length^a^	83	498	487-509	400-626
Adult width^a^	83	194	189-197	148-253
Egg length^a^	160	31.5	30.2-30.8	26.4-36.7
Egg width^a^	160	17.9	17.6-18.2	13.6-23.2
Number of eggs/worm	40	130	120-140	70-231

^a^Measurements are in μm.

Adult *Haplorchis pumilio* worms and eggs obtained the uterus, originating from infected foxes eight weeks post infection, were measured and counted.

**Figure 2:**
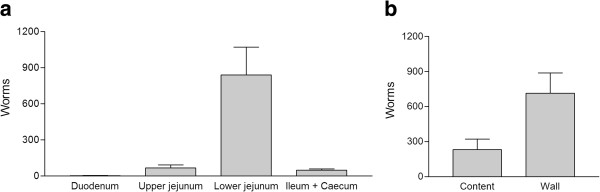
**Worm counts. a**) Distribution of *Haplorchis pumilio *adult flukes in four sections of the small intestine (duodenum, upper jejunum, lower jejunum, ileum + caecum) (mean ± SD) recovered 56 days post infection from eight foxes and **b**) distribution of *H. pumilio *collected from the intestinal content by sieving or incubation and shaking the intestinal wall.

Adult *H. pumilio* worms were recovered from all infected foxes at necropsy as confirmed by morphological examination of stained specimens. Further confirmation was provided by PCR of 9 worms which all had identical sequences which have been uploaded in GenBank with accession numbers JX532156-JX532164. The sequences were 100% identical to the previously reported *H. pumilio*[25]*.* Worm burdens ranged between 116–2070 adult flukes with a mean (± SD) 948 ± 666. The mean establishment percentage of metacercariae was 47% and the range was 6-104%. The number of worms in different parts of the intestine differed significantly (P < 0.0001) with the majority found in the lower part of the jejunum (Figure 2a). Most worms were recovered from the intestinal wall after the shaking and incubation in saline. The EDTA incubations yielded only 3 and 0.04% of the worms after 6 and 24 h, respectively. Significantly more worms were found attached to the mucosa than in the intestinal content (P = 0.024) (Figure 2b). No flukes were found in the liver of any animals and no intestinal flukes were recovered from any of the control foxes.

### Clinical and pathological observations

**Figure 3:**
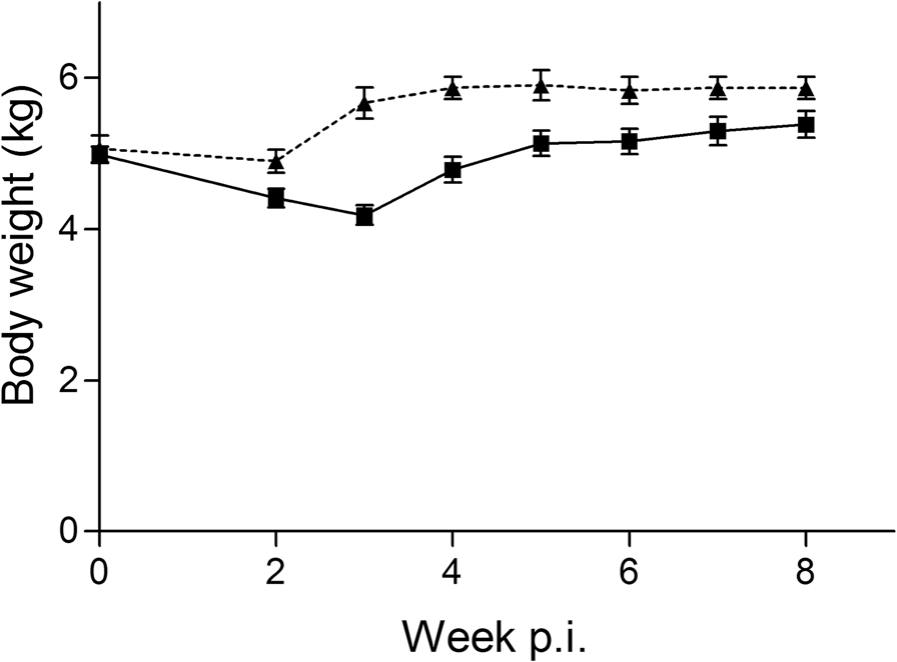
**Live mean body weight of foxes. **The body weight (mean ± SD) of the eight foxes infected with *Haplorchis pumilio *(*black square*) and three uninfected control foxes (*black triangle*) from week 0–8 post infection.

All the infected foxes had anorexia in various degrees starting from day 12 p.i. and lasting for approximately a week. A single fox had severe anorexia for 10 days and had to be fed a salt-sugar solution before it’s appetite returned. The anorexia coincided with a significant loss in body weight in infected versus control foxes as seen in the post hoc analysis in weeks 3–6 (P = 0.0002-0.0353) (Figure 3). No correlation between worm burden and total weight gain was found. No differences in faecal consistency of the infected and control group were found. In general, faeces consistency was on average 2.5 (well-formed with a slightly moist surface) in the first two weeks, above 4 (not well-formed, viscous, diarrhoea) in week 3 and around 3.5 (very moist, but still with some definite form) throughout the rest of the study. The change in faecal consistency was seen after the change in diet (from dry to wet feed) and was similar for both infected and control animals. The mean daily amount of faeces excreted per fox was 95 ± 18 g in the 10-day period. During the days with anorexia the amount of faeces excreted by the infected foxes was substantially reduced, but the amounts were not measured.

**Figure 4:**
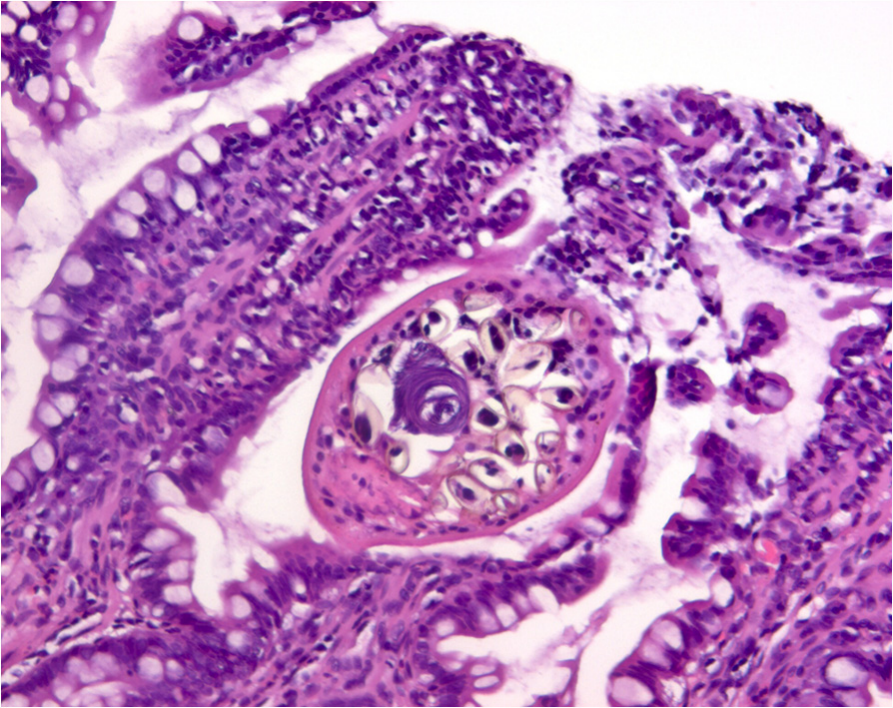
**Histological picture of an adult *Haplorchis pumilio*. **HE-staining of the lower part of jejunum containing an adult *Haplorchis pumilio *worm located in the crypt between two villi.

The macroscopic examination of the intestinal mucosa revealed diffuse hyperaemia and slight oedema in the lower part of jejunum in a single infected fox. In another, five-six petechial bleedings were observed. The livers appeared normal in all foxes. No obvious differences in the HE sections of the jejunum were seen with regard to crypt length, eosinophilic or other inflammatory cell infiltration. An adult worm was found located in the crypt between two villi causing attenuation of the adjacent epithelium at several positions (Figure 4). The worm did not give rise to local eosinophilia or any other apparent cellular response.

### Haematology and biochemistry

**Figure 5:**
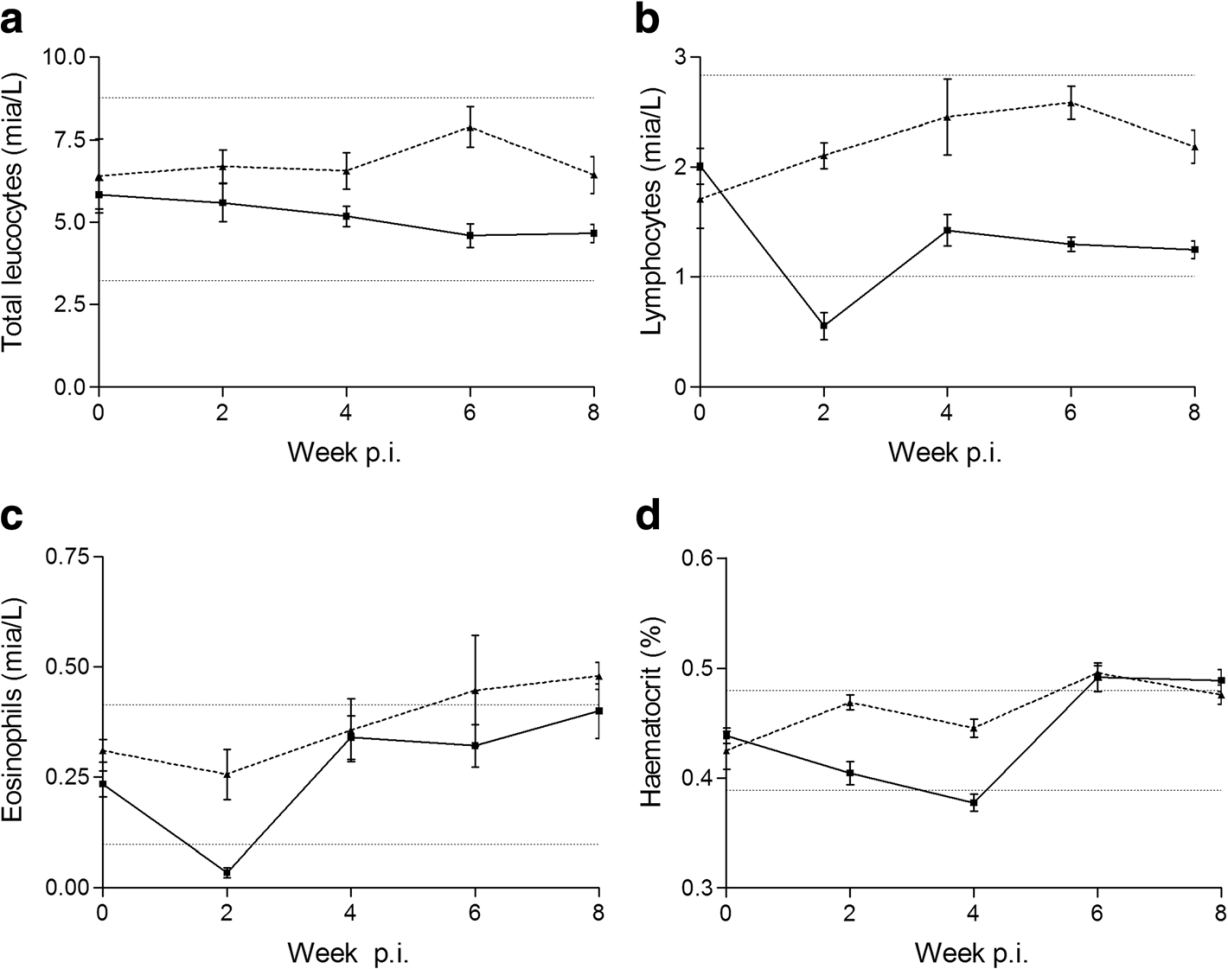
**Haematological findings. a**) Total number of leucocytes (WBC) **b**) lymphocytes **c**) eosinophils **d**) haematocrit (group means ± SD) of eight *Haplorchis pumilio* infected foxes (*black square*) and three uninfected control foxes (*black triangle*) from week 0–8 post infection. Dotted horizontal lines represent a reference range based on mean ± 2 SD for the day 0 measurements (all 11 foxes).

The WBC counts were significantly lower (25%) in the infected group throughout the study period (P = 0.0014) (Figure 5a). The leucocyte differential count in infected foxes showed significantly lower levels (50%) of lymphocytes (Figure 5b) in the whole study period (P < 0.0001) and significantly lower level of eosinophils in week 2 p.i. (P < 0.0001) (Figure 5c) and monocytes in week 6 p.i. (P = 0.013) (data not shown). The neutrophils and basophils did not differ between the groups. In week 2 and 4 p.i. both the total red blood cell count (P =0.0124 and P =0.0001), haemoglobin (P = 0.0094 and P = 0.0002), and haematocrit (P = 0.0003 and P = 0.0002) were significantly lower (15-17%) in infected foxes (Figure 5d – only data for haematocrit shown). Platelet numbers were significantly lower (37%) in the infected group in week 2 (P = 0.0001) (not shown).

**Figure 6:**
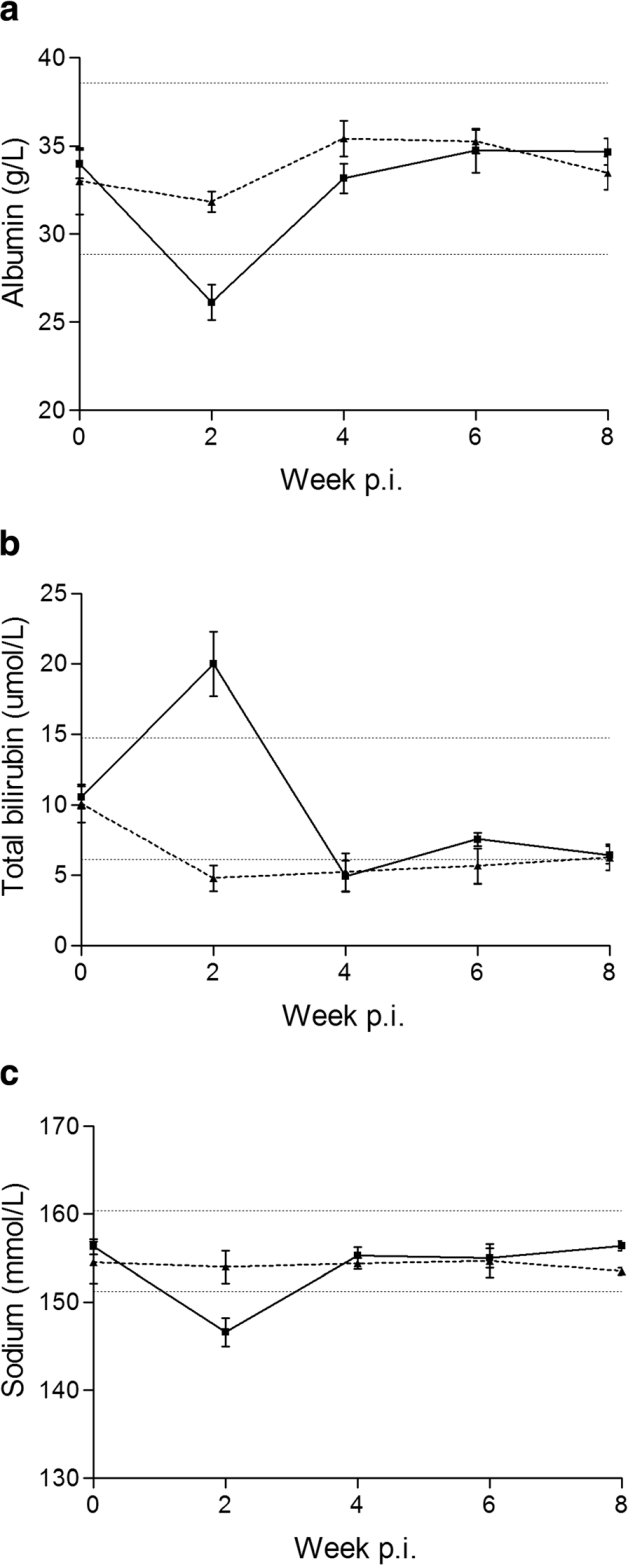
**Biochemical findings in serum. a**) Albumin **b**) Total bilirubin **c**) Sodium (group means ± SD) of the eight *Haplorchis pumilio* infected foxes (*black square*) and three uninfected control foxes (*black triangle*) from week 0–8 post infection. Dotted horizontal lines represent a reference range based on mean ± 2 SD for the day 0 measurements (all 11 foxes).

A significantly lower level of serum albumin (19%) was seen in week 2 p.i. (P = 0.0004) (Figure 6a). A tendency to lower total serum protein was seen in infected foxes in week 2 p.i. as well, but this was not significant. Total bilirubin peaked in week 2 p.i in the infected group and was four times higher than in the controls (P < 0.0001) (Figure 6b). No differences between the groups were seen for GGT or ALT, however, 6 infected animals had ALT levels exceeding the calculated reference values at different time points. Calcium, magnesium, and sodium levels all dropped significantly (15, 23 and 5%, respectively) in week 2 p.i. in the infected group (P < 0.0001, P = 0.0026, P = 0.030, respectively) (Figure 6c – only data on sodium is shown). No clear patterns or major changes in other parameters (fructosamine, alkaline phosphatase, cholesterol, creatinine, phosphate, bile acid, amylase, blood urea nitrogen, and potassium) were found.

## Discussion

This experimental study is the first among other experimental animal models to provide detailed information about the pathogenicity, the fecundity and egg excretion patterns of *H. pumilio* infections.

An acute phase occurred within the first weeks of infection: From week 2 p.i. anorexia occurred in all infected foxes which resulted in weight loss and was associated with a depletion of lymphocytes, eosinophils, and moderate anaemia. Egg excretion also peaked in this initial phase. Appetite, body weight and most haematological parameters were back to normal at the end of the study and egg excretion had found a constant level from day 24 post infection. Important biological information, like finding the pre-patent period to be 9 days and estimating the reproductive capacity to be 58 ± 40 eggs daily per worm, was also gained. The knowledge we obtained on the clinical impact on *H. pumilio* infection in a host relevant to known reservoir hosts in endemic areas can be useful for both clinical management of human infections and in the planning of control programs in aquaculture.

A natural infection pattern with *H. pumilio* is likely to consist of multiple infections over time since farm dogs probably eat raw fish continuously rather than on a single occasion. However, an initial step to describe biological data and host reaction is to use single infections. The infection dose of 2000 metacercariae used for the foxes can be considered at the high end of what, for example, a dog would acquire by a single meal of raw fish based on findings in natural infections in fish. The density of metacercariae in the wild-caught fish from canals and fish from ponds in the Nam Dinh Province in Vietnam was 0.5 and 0.03 metacercariae/g fish tissue [4]. However, metacercariae are not evenly distributed in the fish tissue [26,27] and if a dog happens to eat parts of a fish containing predilection sites for metacercariae, e.g. basis of fins, or several fish or fish parts per day, a dose of 2000 metacercariae is probably not unlikely. Light infections of 500 *H. pumilio* metacercariae in dogs have been found to be asymptomatic [28] but clinical signs have been reported from a study in cats using 80,000 metacercariae of *M. yokogawai*[29] in which infected cats lost their appetite, and became physically inactive during the first two weeks of infection.

This study described a shorter pre-patency period of *H. pumilio*, being only 9 days, than an experimental study in dogs [28]. However, the finding in the foxes is in accordance with detection of gravid *H. pumilio* worms in experimentally infected mice day 9 [9] and the prepatency period of the heterophyid, *Heterophyes heterophyes* reported in humans [30]. Egg excretion in the present study increased rapidly after the infection became patent, peaked on two sampling days, then dropped again and became stable at a level below 500 egp. A similar pattern with an acute phase with high egg output is seen for other trematodes like echinostomes [31]. The peak in egg excretion also coincided with anorexia and hence lower faecal output. A part of the increase could therefore probably be explained by an up-concentration of the eggs in the faeces. Since the total amount of faeces was not measured during this period it was impossible to determine to what extent the increase was attributed to up-concentration or increased egg output by the worms. The number of eggs excreted by the foxes can be considered low compared to the liver flukes (*C. sinensis* 2500–3000 eggs/worm/day) [32] but is comparable to what was estimated for *H. taichui* (82 eggs/worm/day) [33]. Information describing worm burden and reproductive capacity is warranted for developing better diagnostic tools e.g. copro-DNA analysis for detection of minute intestinal flukes [10].

The life expectancy of *Haplorchis* spp. remains unknown but for other heterophyids the life span of the worms is speculated to be less than a year [30]. In our study, all eight foxes harboured adult worms at the time of necropsy and were excreting eggs throughout the 8 week study period. After the initial peak in egg excretion no signs of declining fecundity of the worms were seen. In a previous study with low dose of 500 *H. pumilio* metacercariae in dogs the infection also prevailed until necropsy around 8 weeks p.i. [28]. Hence, a single infection can result in at least two months of patency showing no obvious signs of immune related expulsion of worms; however future studies should aim at describing the host’s immune regulation towards repeated infections mimicking natural circumstances, which might differ from the pattern seen here.

The mean establishment of 47% in this study was considerably higher than in a similar study with dogs (mean 12%) [28] and in an experimental *M. yokogawai* infection in dogs (15-20%) [34]. One might speculate that the metacercariae present in the fish tissue used for infection in this study had a higher infectivity and therefore gave a higher establishment proportion than in studies, where artificially digestion and storage of metacercariae has been performed prior to infection. The high establishment proportion and the persistent egg excretion underline that foxes can serve as a good experimental model for *H. pumilio* infections and perhaps for other zoonotic heterophyids as well [9,35,36].

The predilection site for the flukes being the lower part of the jejunum confirms earlier findings with *H. pumilio*[28]. Whereas previous studies on *M. yokogawai* and *H. heterophyes* described predilection site of the worms in the small intestine to differ somewhat (*M. yokogawai* being found in the duodenum and more anterior than *H. heterophyes* which is located further back [11,29]) both species are reported attached to the mucosa in the crypts of Lieberkühn [11]. The finding of an adult worm in a histological section and the methods for recovering the worms (more than three times as many found by processing the intestinal wall than the content) in the present study also indicated that the majority of the worms were embedded between the villi, probably attached to the mucosa. Case reports with *H. taichui* have shown the worms to be attached to the epithelium with their oral sucker and causing damages like microhaemorrhages and villuos atrophy with eosinophil and lymphocyte infiltration [12]. None of these pathological changes were seen in the present study, nor was any macroscopic changes in the mucosa found. A previous experimental study with *M. yokogawai* in dogs showed that mucosal damage was reversible and that the mucosa returned to a normal four weeks post infection [34]. Future investigations should include necropsies in the early, acute phase of infection to evaluate possible histopathological changes.

Depletion of blood eosinophils was seen in week 2 p.i. but subsequently the level did not differ from the control foxes. The depletion in blood eosinophils in the acute phase of the infection could perhaps be due to an up-regulated, local immune response taking place shortly after infection. Although many helminth infections often are associated with eosinophilia, this is normally not the case for intestinal trematodes [37] and was not the case in this study, either. Hence blood eosinophilia is not a good diagnostic criterion in *H. pumilio* infections in foxes; however, confirmation is needed from other hosts.

The changes in biochemical markers were also mainly seen in week 2 p.i. The decrease in albumin has also been seen in other cases of gastrointestinal diseases and intestinal parasitism [38]. Due to the small size of the protein it is likely to leak into the intestine if the integrity of the mucosa is disrupted. The transient anaemia could be a result of excessive blood cell destruction (haemolysis) or caused by haemorrhages e.g. micro-bleedings caused directly by the flukes. The anaemia was associated with a rise in total bilirubin, the breakdown product of the heme catabolism but this was only seen in week 2 p.i. After a few weeks the acute phase with symptoms declined and most markers analysed in the present study were back to normal. In the infected foxes we observed sporadic increased serum ALT, which is normally used as a marker for hepatic injury in dogs [38]. We are not able to explain these sporadic increases by the fluke infection since no damage was seen in the livers, however, increased ALT activity is known to occur in a wide range of other disorders including hypoxia secondary to anaemia [38], which could perhaps be the case in this instance.

## Conclusion

To conclude, this study reported fundamental biological aspects of a *Haplorchis pumilio* infection: a pre-patent period of 9 days, an initial peak followed by a constant output of eggs and high establishment proportions. These findings should be taken into consideration when drug control programs of the dog reservoir host are to be established. The study also described the clinical impact of infection and documented that an acute phase with anorexia, transient weight loss and anaemia, lowered lymphocyte, eosinophil count and serum-albumin takes place. Hence, a *H. pumilio* infection should not be regarded as always asymptomatic [39]. Hopefully this knowledge will encourage future studies in both domestic animals and humans to increase the understanding of both single and continuous exposures to minute intestinal fluke infections.

## Abbreviations

FZT: Fish-borne zoonotic parasites; p i: Post infection; epg: Eggs per gram; SD: Standard deviation; WBC: Total white blood cell count; RBC: Total red blood cell count; ALT: Alanine aminotransaminase; GGT: Gamma-glutamyltransferase; ANOVA: Analysis of variance; EDTA: Ethylenediaminetetraacetic acid; FIBOZOPA: Fish-borne Zoonotic Parasites.

## Competing interests

The authors declare no competing interests.

## Authors’ contributions

SN, SMT, AD and MVJ conceived and designed the study. SN planned the study, performed the parasitological examinations on faecal samples and adult worms and did the statistical analysis. PWK performed the molecular work and analysis and drafted the sections about the molecular findings. PSL, MVJ and SN performed the histo-pathological examinations. PSL drafted the sections on the histo-pathological findings. SN, SMT, AD and MVJ drafted the remaining parts of the manuscript. All authors read and approved the final manuscript.
